# 

**Published:** 1904-01-02

**Authors:** 


### Publications.

### MESSRS. THOS. AGNEW & SONS' ENGRAVINGS.

### Re "THE DOCTOR" By LUKE FILDES, R.A.

### WARNING.

NOTICE IS HEREBY GIVEN that copies of several Catalogues issued by American Electro-type Manufacturing Firms are in circulation in the United Kingdom in which offers are made to supply Electro-types of the above copyright Picture, an Engraving of which is published by Messrs. Thos. Agnew & Sons.

THE IMPORTATION into the United Kingdom of these Catalogues or of such Electro-types is a breach of the Copyright Acts.

NOTICE IS FURTHER GIVEN that proceedings will be immediately instituted against all persons found importing, selling, exhibiting, or in any way dealing with the said Catalogues and Electro-types or any unlawful copies of the said picture.

Guildhall Yard, E.C., 1st January, 1904.

WATKIN WILLIAMS, GRAY & STEEL,
Solicitors for Messrs. Thos. Agnew & Sons.

---

### THE HANDBOOK OF THE OPEN-AIR TREATMENT

By Dr. CHARLES REINHARDT

(Visiting Physician to Hailey Sanatorium, Wallingford, and Stourfield Park Sanatorium, Bournemouth),

Contains Views, Descriptions, and Information respecting 30 Sanatoria in Great Britain.

Price: Cloth, 2/6; Paper, 1/- (postage 4d.)

The "HEALTH RESORT" Publishing Office, 379 Strand, London, W.C.

---

JUST ISSUED. EIGHTH EDITION. Crown 8vo, 10s. 6d.

### PHARMACY, MATERIA MEDICA AND THERAPEUTICS

Is now rewritten and printed in large type, and is made a companion to the following volume. It contains an account of the action **of every drug in the B.P.**, as well as a section upon all the newest drugs, with an Introduction to the Art of Dispensing. With woodcuts, prescriptions, &c.

Also FOURTH EDITION. 1055 pages, 8vo. 16s.

### DICTIONARY OF TREATMENT.

Containing a Revised and up-to-date Article upon every Diseased Condition, whether Medical, Surgical, Gynæcological, or Ophthalmological, as well as Articles upon Poisoning, Drowning, and the various Surgical Emergencies and Accidents, arranged for Rapid Reference.

By W. WHITLA, M.A., M.D., Professor of Materia Medica, Queen's College, Belfast; Senior Physician to the Royal Victoria Hospital, &c.

The Publisher will supply both volumes, carriage paid, for 21/-

London: HENRY RENSHAW, 356 Strand.

---

Crown 8vo. Illustrated. Price 2s. 6d. net.

### ANÆSTHETICS. By J. BLUMFELD,

M.D.Cantab., Senior Anæsthetist to St. George's Hospital; Anæsthetist to the Grosvenor Hospital for Women and Children, London, and to the National Dental and Metropolitan Hospitals.

*Lancet.*—"....In writing this handbook the author has been quite successful." *Australasian Medical Gazette.*—"We can strongly commend this book." *Medical Chronicle.*—"The book is one which we can heartily recommend to senior students and practitioners."

London: BAILLIÈRE, TINDALL & COX, 8 Henrietta St., Covent Garden.

---

Just published. Sixth Edition, thoroughly Revised, with 4 Plates and 166 Illustrations, Crown 8vo., 10s 6d.

### DISEASES OF WOMEN. A Practical Text-book.

By Arthur H. N. LEWERS M.D.Lond., F.R.C.P.Lond., Senior Obstetric Physician to the London Hospital, and Lecturer on Midwifery in the London Hospital Medical School; Examiner in Obstetric Medicine to the University of London; Examiner in Midwifery and Diseases of Women at the Conjoint Board of the Royal College of Physicians of London and of the Royal College of Surgeons of England, &c.

[*Lewis's Practical Series.*

By the same Author, with 3 Coloured Plates and 51 Original Illustrations, 8vo., 10s 6d. net

### CANCER OF THE UTERUS: A CLINICAL MONOGRAPH ON ITS DIAGNOSIS AND TREATMENT.

With the AFTER-RESULTS in SEVENTY-THREE CASES treated by RADICAL OPERATION.

"The illustrations form a valuable feature of the book. An inspection of the figures alone would give a clear and fairly complete idea both of the macroscopic and microscopic appearances of malignant disease as it affects the uterus."—*Medical Chronicle.*

LONDON: H. K. LEWIS, 136 Gower Street, W.C.

### Publications—*continued.*

Second Edition. Crown 8vo., price 3/6 net.
With original "Plea—1900—for the State Registration of Nurses."

**MOCK NURSES OF THE LATEST FASHION.**

By FREDERICK J. GANT, F.R.C.S.,
Consulting Surgeon to the Royal Free Hospital.

BAILLIÈRE, TINDALL & COX,
Henrietta Street, Covent Garden, London.

NOW READY. Demy 8vo. Price 5/- net.

**ASTHMA IN RELATION TO THE NOSE.**

*WITH NOTES OF 402 CASES.*

By ALEXANDER FRANCIS, M.B., B.C.Cantab.

London: ADLARD & SON, Bartholomew Close, E.C.

### NOTICE TO CORRESPONDENTS.

All MSS., Letters, Books for Review, and other matters intended for the Editor should be addressed THE EDITOR, "THE HOSPITAL," THE HOSPITAL BUILDINGS, 28 & 29 SOUTHAMPTON STREET, STRAND, W.C.

The Editor cannot undertake to return rejected MS., even when accompanied by stamped directed envelope.

**Notice to Advertisers and Subscribers.**

All Advertisements, Orders for Copies of THE HOSPITAL, and Business Communications should be addressed to **THE MANAGER (Not to the Editor),** THE HOSPITAL, 28 & 29 Southampton Street, Strand, London, W.C.

The Cost of Subscription, post free, is as follows:—Per week, 3½d.; per quarter, 3s. 9d.; per half-year, 7s. 6d.; per annum, 15s. Foreign and Colonial:—Per annum, 19s. 6d., payable, in all cases, in advance.

**NOTICE.—Vol. XXXIV. of "The Hospital" (April 1903 to September 1903), in handsome cloth binding, is now on sale at the office of this Journal, price 10s. 6d. Cases for binding the Numbers contained in this Volume 2s. Index to Vol. XXXIV., 2½d., post free.**

**The Monthly part for January is now ready. Price 1s., by post, 1s. 3d.**

### Tendencies to Consumption:

### HOW TO COUNTERACT THEM.

**By J. D. E. MORTIMER,**

M.B. Lond., M.R.C.S., L.S.A.,

Author of "Home Nursing of Sick Children."

Crown 8vo. cloth, **2/6** post free.

*Of all Booksellers, or of*

THE SCIENTIFIC PRESS, Ltd., 28 & 29 Southampton Street, Strand, London, W.C.

### MEDICAL APPOINTMENTS.

**Dr. N. H. Alcock**, Lecturer on Physiology at University of London Medical School. **H. Case**, L.R.C.P.&S.Edin., L.F.P.S. Glasg., Certifying Factory Surgeon for the Withnell District, County Lancaster. **W. T. Crawford**, M.D.Edin., Medical Officer of the Kilton Hill Infirmary of the Worksop Union. **J. R. Edward**, M.B., Ch.B., House-Physician to the Nottingham General Hospital. **C. H. Fagge**, F.R.C.S., M.S.Lond., Surgeon in charge of In-patients to the Evelina Hospital for Sick Children, Southwark. **W. S. Hall**, M.B., Ch.B.Aberd., District Medical Officer of the Bradford Union. **C. Haslewood**, M.B., C.M.Aberd., District Medical Officer of the Clitheroe Union. **Ernest E. U. Hawkes**, L.S.A., Senior Resident Medical Officer at the Nottingham Union Workhouse. **N. N. A. Houghton**, M.B.Lond., Assistant House-Physician to the Nottingham General Hospital. **Thomas Lumsden**, M.D., Registrar to Out-patients, Central London Throat Hospital. **W. Hunter Richards**, M.B., M.S., F.R.C.S., Honorary Gynæcologist to the Plymouth Public Dispensary. **R. C. Verley**, M.B., Ch.B.Edin., Honorary Surgeon to the Swansea Hospital.

KING'S COLLEGE HOSPITAL.—The following resident appointments have been made:—Senior House-Physician: **R. H. Lee**, M.R C.S., L.R.C.P. Junior House-Physician: **W. B. Clarke**, M.R.C.S., L.R.C.P. House-Accoucher: **W. C. Smales**, M.R.C.S., L.R.C.P. Assistant House-Accoucher: **Cyril Cheatle**, M.R.C.S., L.R.C.P. House-Surgeons: **J. A. Drake**, M.B.Lond.; **E. U. Williams**, M.R.C.S., L.R.C.P.; **N. E. Dunkerton**, M.R.C.S. L.R.C.P.

**"The Hospital" Institutional Library.**

"Hospitals and Asylums of the World." 4 Vols., with a Portfolio of Plans, complete £12 12s.

"Burdett's Hospitals and Charities, 1903." 5s. net.

"Burdett's Official Nursing Directory, 1903." 3s. net.

"Cottage Hospitals, General, Fever, and Convalescent." 10s. 6d.

"Uniform System of Accounts for Hospitals and Public Institutions." New Edition. 4s. net.

"Hospital Expenditure: The Commissariat." 2s. 6d.

"A Legal Handbook for the Use of Hospital Authorities." 2s. 6d.

"The Cottage Hospital Case Book or Register of Patients." 10s. and 12s. 6d.

All these are published by the SCIENTIFIC PRESS, Ltd., and may be obtained through any bookseller, or direct from the publishers, 28 and 29 Southampton Street, Strand, London, W.C.

### Medical Appointments.

### Hints on How to Start a District Nursing Association.

BY A COUNTY SUPERINTENDENT.

Price 1/1 post free.

This work, which is the outcome of great personal experience, gives full particulars on how to start a District Nursing Association, and will be found of the greatest value to those who desire to establish such an institution in their own immediate neighbourhood.

The information it contains will also prove exceedingly useful to all in any way interested in District Nursing.

*Of all Booksellers, or of*

THE SCIENTIFIC PRESS, LTD., 28 & 29 Southampton Street, Strand, London, W.C.

### Two Useful Text=books.

**ELEMENTARY ANATOMY AND SURGERY FOR NURSES.**

By WILLIAM McADAM ECCLES, M.S. Lond., M.B., F.R.C.S., L.R.C.P.

Crown 8vo., profusely illustrated, cloth, **2/6** post free.

"The book will be valuable to nurses who are beginning the study of anatomy. The instruction is given very clearly and concisely, and the reader is able to glean much information in a very condensed form."—*Nursing Notes.*

**ELEMENTARY PHYSIOLOGY FOR NURSES.**

By C. F. MARSHALL, M.D., B.Sc., F.R.C.S.

Crown 8vo., illustrated, cloth, **2/-** post free.

"Nurses will find in it all that they require to know of the subjects treated."—*Daily Chronicle.*

*To be obtained of all Booksellers, or of*

THE SCIENTIFIC PRESS, LTD., 28 & 29 Southampton Street, Strand, London, W.C.

### THE NURSING PROFESSION: How and Where to Train.

**By Sir HENRY BURDETT, K.C.B.**

A reliable and up-to-date Guide to Training for the calling of a Nurse. In its pages would-be Nurses will find all the information they require.

New Edition. Price 2/-, post free 2/4.

Of all Booksellers or of THE SCIENTIFIC PRESS, LTD., 28 & 29 Southampton Street, Strand, London, W.C.

### Some Interesting Books for New Year's Gifts = = = = = = =

### THE MYSTERY AND ROMANCE OF ALCHEMY AND PHARMACY.

By C. J. S. Thompson. Price **5/-** post free.
A fascinating book with many quaint illustrations.

### POISON ROMANCE AND POISON MYSTERIES.

By C. J. S. Thompson. Price **2/6** post free.
An absorbing and exciting work.

### FROM A NURSE'S NOTE-BOOK.

By Honnor Morten. Price **2/-** post free.
Sketches from a Nurse's Experience. Powerfully written and full of interest.

### GEORGE HARLEY, F.R.C.S., OR THE LIFE OF A LONDON PHYSICIAN.

By Mrs. Alec Tweedie. Price **6/-** net; **6/5** post free.
A delightful memoir.

### MEDICAL HISTORY FROM THE EARLIEST TIMES.

By E. T. Withington. M.A. Price **12/6** net; **12/10** post free.
Most interesting, accurate, and well written.

### SOME HEALTH ASPECTS OF EDUCATION.

By Dr. Percy Lewis. Price **1/-** post free.

### THE HUMAN BODY: Its Personal Hygiene and Practical Physiology.

By Professor Colton, of Illinois University. Price **5/-** post free.
A most fascinating and thorough treatise. Profusely illustrated.

### CHATS ABOUT THE MICROSCOPE.

By H. C. Shettey. Price **1/-** post free.
The story of the microscope told in simple language.

### FIRST AID TO THE INJURED AND MANAGEMENT OF THE SICK.

By E. J. Laylers, M.D. Price **3/6** post free.
An Ambulance Handbook and Elementary Manual of Nursing.

*These books are obtainable of all Booksellers, and are published by*
THE SCIENTIFIC PRESS, LIMITED, 28 & 29 Southampton Street, Strand, London,
*who will send their latest Catalogue of Nursing Text-books post free to any Nurse on application.*

